# Retaining Healthcare Workers: A Systematic Review of Strategies for Sustaining Power in the Workplace

**DOI:** 10.3390/healthcare11131887

**Published:** 2023-06-29

**Authors:** Neeltje De Vries, Olivia Lavreysen, Anke Boone, José Bouman, Szymon Szemik, Kamil Baranski, Lode Godderis, Peter De Winter

**Affiliations:** 1 Department of Internal Medicine, Spaarne Gasthuis, P.O. Box 417, 2000 AK Haarlem and Hoofddorp, The Netherlands; 2 Spaarne Gasthuis Academy, P.O. Box 417, 2000 AK Haarlem and Hoofdorp, The Netherlands; 3 Centre for Environment and Health, University of Leuven (KU Leuven), P.O. Box 952, 3000 Leuven, Belgium; 4 Department of Epidemiology, School of Medicine in Katowice, Medical University of Silesia, 40-055 Katowice, Poland; 5 IDEWE, External Service for Prevention and Protection at Work, 3000 Leuven, Belgium; 6 Department of Paediatrics, Spaarne Gasthuis, P.O. Box 417, 2000 AK Haarlem and Hoofddorp, The Netherlands; 7 Leuven Child and Health Institute, University of Leuven (KU Leuven), P.O. Box 3717, 3000 Leuven, Belgium; 8 Department of Development and Regeneration, University of Leuven (KU Leuven), P.O. Box 611, 3000 Leuven, Belgium

**Keywords:** healthcare workers, physicians, nurses, retention, systematic review, interventions, personnel turnover

## Abstract

The shortage of healthcare workers is a growing concern. The COVID-19 pandemic and retirement wave have accelerated turnover rates. This systematic review aimed to identify and analyse the existing interventions for job retention of healthcare workers, in terms of nurses and physicians, in a hospital setting. A comprehensive search was conducted within three electronic databases, guided by the preferred reporting items for systematic review and meta-analyses (PRISMA) and synthesis without meta-analysis (SWiM) guidelines, this resulted in 55 records that met the inclusion criteria. The intervention outcomes are categorized into substantial themes: onboarding, transition program to a different unit, stress coping, social support, extra staffing, coping with the demands of patient care, work relationships, development opportunities and department resources, job environment, work organization, recruitment approach, and technological innovations. Considering the literature, onboarding programs and mentoring for nurses and physicians are recommended. Additionally, other interventions described in this review could positively affect the retention of nurses and physicians. When selecting an intervention for implementation, managers and human resources should consider the intervention that matches the determinant of intention to leave of their healthcare workers and the hospital’s mission, vision, and values. Sharing the success stories of implemented interventions may benefit healthcare organizations.

## 1. Introduction

Worldwide, there is a growing concern about the number of healthcare workers, which currently suffers from a shortage of 5.9 million nurses [1] and 4.3 million doctors [2]. Turnover rates were accelerated by the COVID-19 pandemic. For example, a study in the United States revealed that 18% of healthcare workers left their jobs as a result of the pandemic [3]. Furthermore, the outflow of healthcare workers leaving the hospital will also increase in the future with the retirement of healthcare workers. Globally, about 17% of all nurses are expected to retire within the next ten years. In particular, the ageing workforce in the United States and Europe means that retirement rates will remain high over the next ten years [1].

Furthermore, the healthcare system is struggling to recruit the younger generation of healthcare workers who deem the nursing profession unattractive due to salary or low job status [4] and physicians deem the medical profession due to a lack of training positions and the lack of salary comparing to their working conditions [5,6]. These arguments for why younger generations of nurses and physicians are less willing to start a healthcare career also explain the push and pull factors resulting in the international migration of healthcare personnel. Lots of physicians are mentioning working in high-income countries and strained healthcare systems such as Australia, New Zealand, and Central Asia, instead of low- or middle-income countries or less-strained countries (e.g., Ireland, the United Kingdom, or sub-Saharan Africa) [7,8,9]. This migration process results in an enlarging shortage of physicians in these countries with a tremendous shortage of doctors [7,8].

Altogether, this looming crisis demands a coordinated response with the government, health organizations, and other stakeholders working together to ensure that healthcare workers have the support they need to remain in the field.

The turnover rate of nurses and physicians poses substantial financial and non-financial burdens for healthcare organizations [10]. Multiple studies have found an association between nurse staff turnover and patient outcomes such as patient health [11], length of stay of hospitalized patients [12], and quality of care [13]. Physician turnover has also been shown to affect patient care costs by disrupting the continuity of care and causing dissatisfaction in patients who have lost their current provider or the need to establish a new relationship with another provider [14]. Moreover, high turnover rates reduce staff productivity because there is limited personnel to complete the tasks [15]. This can lower the morale of the remaining staff [16,17] and may lead to additional turnover among the remaining employees [14]. As a result, healthcare organizations incur enormous costs associated with recruiting, hiring, and instructing new personnel [18,19]. In the United States, the recruitment cost per nurse vacancy has been estimated between USD 10,000 to USD 88,000 [18], while costs for physician recruitment are even higher, ranging from USD 88,000 to USD 1,000,000 per physician [14,19,20].

Aside from the financial problems caused by turnover, frequent staff turnover can decrease the job satisfaction of healthcare workers and trigger them to leave the profession. In addition, this process results in a loss of knowledge and experience in the healthcare profession [14,21].

In view of the many problems associated with turnover, it is crucial to minimize the impact of the shortage of nurses and physicians by retaining them in their hospital. Furthermore, retaining nurses and physicians will improve patient health, length of stay, and quality of care. However, an overview of interventions which are effective for retaining nurses and physicians in hospitals is lacking. To address this issue, this systematic review aims to identify and analyse the current interventions that minimize nurse and physician job retention in a hospital setting.

## 2. Method

This systematic review constitutes the starting point of an EU-funded project named METEOR (MEnTal hEalth: fOcus on Retention of healthcare workers) [22].

### 2.1. Design and Population

The systematic review was carried out in accordance with the Preferred Reporting Items for systematic review and meta-analysis (PRISMA) statement [23] and the synthesis without meta-analysis (SWiM) reporting guidelines [24]. PRISMA checklist and SWiM items can be found in Supplemental S1. At the international prospective register of systematic reviews (PROSPERO), the systematic review has been recorded, CRD42022364748.

To create homogeneity in the results, the population studied in this review included healthcare professionals in terms of nurses and physicians in a hospital setting.

### 2.2. Data Sources and Searches

The conducted literature search string in this systematic review was identical to the earlier published systematic review of De Vries et al. [25]. De Vries et al. [25] used the outcomes including determinants impacting retention, whereas this current study included studies on how to improve retention. The design of the search string was set up using the domain, determinant and outcome framework. The domain contained the following synonyms: ‘health personnel’, ‘healthcare workers’, ‘healthcare providers’, ‘healthcare professionals’, ‘health workforce’ and ‘health workers’, ‘nurses’, ‘nurse’, ‘nursing personnel’, ‘physicians’, ‘physician’ or ‘doctor’. Synonyms for the domain were ‘determinants’, ‘factors’, ‘predictors’, and ‘interventions’. As outcomes, the following terms were used: ‘personnel turnover [Mesh]’, ‘personnel turnover’, ‘retaining personnel’, ‘job retention’, ‘retention rates’, ‘turnover intention’, ’intention to leave’, ‘intention to quit’, ‘intention to stay’. The synonyms in selecting domain, determinant, and outcome were combined with OR. The overall domain-, determinant-, and outcome sections were combined with AND [25]. The entire search string is consultable in Supplemental S2.

The search string was developed in Cinahl, Embase, and PubMed in the week of 18 July 2022 [25].

### 2.3. Screening and Data Extraction

Articles were included if they were conducted between 2012 and July 2022 and if the intervention was applied to healthcare workers, namely nurses and physicians. The included manuscript must be written in English, and the research must be conducted in a hospital setting. Study designs such as systematic reviews, thesis, guidelines, and study protocols were excluded. Furthermore, the study was excluded if the full text was unavailable. There were no restrictions in sampling choice. After screening the title and abstract the full texts were studied. Three pairs of two independent reviewers (AB, KK, OL, SS, NdV, and PdW) conducted the screenings.

Furthermore, quality assessment was conducted using the Mixed Methods Appraisal Tool (MMAT) version 2018. The MMAT was selected because of the heterogeneity of study designs included in this systematic review. The same pair of reviewers conducted the quality assessment independently to decrease the change of bias. Disagreement about study eligibility was resolved through consensus discussion or by an extra author, not a duo member. To show an overview of the quality of included articles, a quality rating was calculated showing an overall score. Answering ‘Yes’ in the MMAT tool counted for one point, whereas answering ‘No’ counted for zero points in the overall score. If a quality criterion was answered with ‘Cannot tell’, more information was needed to give a legit answer in terms of ‘Yes’ or ‘No’ [26] and the criterion was not included in the overall score. The final overall score is an overview. An overall score of zero points is labelled as a bad-quality study. All other scores are labelled as non-bad quality studies. The overall score of the quality assessment does not reveal what aspect of the assessment is questionable [27]. Therefore, it is desirable to scale the overall score with the complete quality assessment screening, which will be shown in Supplemental S3.

Data were extracted into multiple characteristics: type of study, country, type of healthcare worker (physicians or nurses), sample size, the department where the intervention took place, description of the intervention, and results on the micro-level, meso-level and macro-level. Micro-level: refers to the individual level of analysis, such as a person’s behaviour. Meso-level: refers to the study of groups of people and their interactions, such as organizations and communities. Macro-level: refers to the study of large-scale phenomena and the broader forces that shape society, such as political, economic, and cultural systems. Furthermore, the factors that influence the effectiveness of the intervention were described, and an additional check was done on whether a price analysis was conducted.

Due to the heterogeneity between studies regarding the study designs and outcome measures, a meta-analysis was not conducted.

## 3. Results

**Figure 1:**
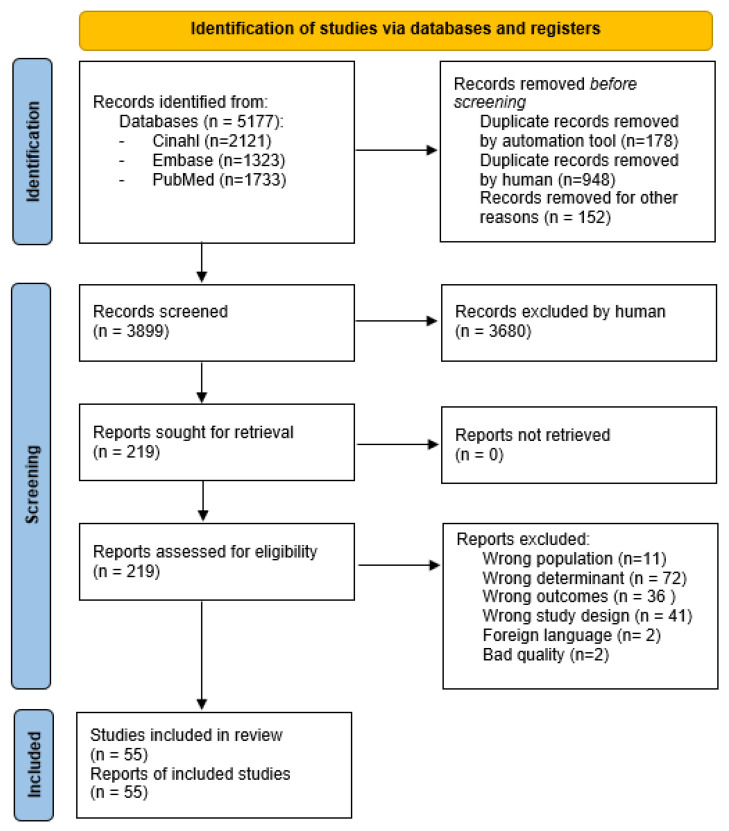
PRISMA flow diagram.

The literature search resulted in 5177 articles. Before screening 1126 duplicates were removed and 178 duplicates were detected automatically by an application. The detected duplicates were checked by the author and removed by hand. The remaining papers were checked by hand for any missed duplicates by the application. This resulted in 948 extra duplicate papers which were removed. Moreover, 152 records were removed due to foreign language. In total, 3899 records were screened on inclusion and exclusion criteria, and 219 documents were assessed for eligibility. After reading full texts, 162 records were excluded for not fitting the inclusion or exclusion criteria. Two records were excluded due to bad quality [28,29]. For full quality assessment, Supplemental S3 can be consulted. Finally, 55 records were included in this systematic review (Figure 1).

### 3.1. Methodological Characteristics of the Studies

Of the included records, 85.5% (*n* = 47) were quantitative research, 9% (*n* = 5) were qualitative research, and 9% (*n* = 5) were mixed-method studies. Of the included studies, 83.6% (*n* = 46) focused on nurses, 7.2% (*n* = 4) on physicians, 3.6% (*n* = 2) on both, and 7.2% (*n* = 4) on others (including nurses and physicians). Most studies were completed in the US (43.6%) or Asian (27.3%) countries. The quality of records differed, as shown in Table 1.

**Table 1:** Data-extraction table and quality assessment summary of included records.

First Author (Year)	Type of Study	Country	Sample	Sample Size	Department	Intervention	Quality Assessment ^a^
Adams, A. (2019) [30]	Cross-sectional	US	Nurses	38	ER	Cultural Change Toolkit	3\4
Al Sabei, S.D. (2022) [31]	Descriptive cross-sectional	Oman	Nurses	2113	Multiple	Interprofessional teamwork	5\5
Alvaro, C. (2016) [32]	Pretest-posttest	Canada	Other	158 patients, 367 staff	Complete hospital	The architectural design of the hospital	5\5
Arora, R. (2017) [33]	Retrospective	Thailand	Physicians	19,338	Multiple	Special Rural Recruitment track	3\4
Aull, M. (2022) [34]	Descriptive study	US	Nurses	Unknown	Unknown	The Academic Partnership Program	1\1
Baik, D. (2019) [35]	Cross-sectional	US	Nurses	66	Cardiothoracic surgery	Interprofessional team intervention	4\5
Baillie, L. (2019) [36]	Case study design	UK	Nurses	22	Geriatric ward	Shift Length	4\5
Blegen, M.A. (2015) [37]	Longitudinal randomized multisite design	US	Nurses	678	Newly graduates	Transition-to-practise Program	3\4
Brabson, L.A. (2019) [38]	Cross-sectional	US	Physicians	100	Psychiatric outpatient clinic	Three EBP training models	4\4
Brewer, C.S. (2012) [39]	Longitudinal panel design	US	Nurses	1653	NA	Magnet hospital	2\3
Çamveren, H. (2022) [40]	One group pretest-posttest	Turkey	Nurses	56	Internal, surgical and ICU	Organizational socialization model-based preceptorship program	4\5
Chang, H.Y. (2021) [41]	Adopted two-wave study design	Taiwan	Nurses	331	Unknown	Robots	5\5
Chen, S. (2021) [42]	Longitudinal cohort	US	Nurses	293	ER, ICU and general ward	Adaptive education program	4\4
Chu, X. (2022) [43]	Time-lagged research design	China	Nurses	234	Unknown	Nurses’ strength	4\5
Concilio, L. (2021) [44]	RCT	US	Nurses	21	Unknown	6-week digital intervention text messaging	2\3
Daniels, F. (2012) [45]	Longitudinal Cohort	US	Nurses	Unknown	Unknown	70% Full-Time Commitment	3\4
Dawood, M. (2019) [46]	Interviews	UK	Nurses	12	ER	Dual roles	4\4
Dawson, A.J. (2014) [47]	Interviews	Australia	Nurses	362	Medical, surgical ward	Providing employment options, rewarding performance, enhancing professional development, and training, and improving management practice.	3\3
Deng, J. (2019) [48]	Mixed method	China	Other	572 Health care personnel	Multiple	Comprehensive reform of the hospital	5\5
Duffield, C. (2018) [49]	Cross-sectional	Australia	Nurses	154	Acute Care	Adding unregulated nurses support workers to existing nurse staffing	5\5
El Khamali, R. (2018) [50]	RCT	France	Nurses	198	ICU	A five-day stress-coping course	5\5
Fleig-Palmer, M. (2015) [51]	Cross-sectional	US	Physicians	159	Acute Care	Interpersonal mentoring	3\4
Fleming, P. (2012) [52]	Retrospective	Canada	Physicians	391	Unknown	Provisional licensing to attract International Medical Graduates physicians	3\3
Forde-Johnston, C. (2022) [53]	Mixed method	UK	Nurses	576	Acute Care setting	Listening to Staff events (L2S)	3\4
Gilroy, H. (2020) [54]	Descriptive	US	Nurses	35	Paediatrics	The Bridge Program	2\4
Guo, Y.F. (2020) [55]	RCT	China	Nurses	73	Medicine, Surgical and others	WeChat 3GT	3\3
Harris, K.K. (2017) [56]	Mixed method	US	Both	47	Acute post-surgical oncology unit	Combination of multiple communication strategies.	1\2
Hernandez, S.H.A. (2020) [57]	Retrospective longitudinal cohort study	Mexico	Nurses, new graduates	472	Unknown	UNM CON/UNMH Internship program for newly graduated RN	4\5
Hines, M. (2019) [58]	Quasi-experimental	US	Nurses	16	New-born department	American Nurses Association’s self-care guidelines	3\5
Huang, T.L. (2022) [59]	Observational study	Taiwan	Nurses	331	Unknown	Effort Ensuring Smooth Operation (EERSO)	5\5
Im, S.B. (2016) [60]	RCT	Korea	Nurses, new graduates	49	Unknown	The Huddling Program	4\4
Jensen, C.L. (2021) [61]	RCT	US	Other	130	Unknown	Facility dogs	5\5
Kaihlanen, A.M. (2020) [62]	Cross-sectional survey study	Finland	Nurses, new graduates	712	Unknown	The final clinical practicum experience	4\4
Kang, C.M. (2016) [63]	Mixed method	South-Korea	Nurses, new graduates	17	Unknown	Situational Initiation Training Program (SITP)	5\5
Kang, J. (2019) [64]	Cluster quasi-randomized trial	South-Korea	Nurses	72	Unknown	A cognitive rehearsal intervention (smartphone application)	4\4
Kang, J. (2017) [65]	RCT	Sweden	Nurses	40	Multiple	Cognitive rehearsal program	5\5
Kester, K.M. (2020) [66]	Longitudinal cohort	US	Nurses	338	Thoracic surgery	Prospective Staffing Model	4\4
Koneri, L. (2021) [67]	Cohort study	US	Nurses, new graduates	50	New graduates	One-year residency program using touchpoints	4\5
Kullberg, A. (2016) [68]	Quasi-experimental	Malaysia	Both	58 nurses, 2 physicians	Oncology	Fixed scheduling	4\5
Melnyk, B.M. (2021) [69]	Cross-sectional descriptive	US	Nurses	2344	Unknown	The Advancing Research and Clinical practice through close Collaboration (ARCC) Model	5\5
Mohamadzadeh Nojehdehi, M. (2015) [70]	Descriptive comparative design	Iran	Nurses	248	Unknown	The excellence program	3\3
Morphet, J. (2015) [71]	Mixed method	Australia	Nurses	118	ER	Transition to Specialty Practice Program (TSPP)	5\5
Moss, M. (2022) [72]	Randomized trial	US	Other	165	Unknown	Creative arts therapy (CAT) programs	4\5
Rudin, N.M.N. (2018) [73]	Cross-sectional	Malaysia	Nurses	61	Multiple	Mentorship Program (MNMSN)	3\3
Rushton, C.H. (2021) [74]	Longitudinal pretest-posttest design	US	Nurses	415	Unknown	Mindful Ethical Practice and Resilience Academy (MEPRA)	4\4
Schroyer, C.C. (2020) [75]	Quasi-experimental	India	Nurses, new entering	70	Specialty unit within critical care service	AMSN Mentoring Program	5\5
Tang, Y. (2022) [76]	Quasi-experimental	Taiwan	Nurses	24	Multiple	Humanoid Diagram Teaching Strategy (HDTS)	5\5
Tseng, C.N. (2013) [77]	Quasi-experimental	Taiwan	Nurses, new graduates	42	Unknown	Externship program (EP) compared to. Corporate-academic cooperation program (CACP)	4\4
Vardaman, J.M. (2020) [78]	Cross-sectional	US	Nurses	257	Medical/surgical	Change-related self-efficacy (CSE)	3\3
Walker-Czyz, A. (2016) [79]	Retrospective analysis	US	Nurses	Unknown	Medical surgery and critical care	Integrated Electronic Health Record (EHR)	2\2
Williams, F.S. (2018) [80]	Retrospective, cross-sectional	US	Nurses, new graduates	3484	Unknown	One-to-one and group mentoring on transition to practice	4\4
Winslow, S. (2019) [81]	Cross-sectional	US	Nurses	39	Magnet hospital	Partnership model of care delivery	2\3
Wright, C. (2017) [82]	Descriptive pretest-posttest	US	Nurses	1497	Magnet hospital	Self-scheduling	2\3
Zhang, Y. (2019) [83]	Longitudinal, non-randomized control study	China	Nurses, new graduates	199	Unknown	One-on-one mentorship program	5\5
Zhong, X. (2021) [84]	Randomized trial	China	Nurses	68	Paediatrics	A humanistic care teaching model	4\5

^a^ The quality assessment was conducted using the Mixed Methods Appraisal Tool (MMAT) (version 2018). ‘Yes’ counted for one point and ‘No’ for zero points. In case a quality criterion was answered with ‘cannot tell’, more information was needed to give a legit answer in terms of ‘yes’ or ‘no’ [26]. Therefore, this criterion is not included in the overall score.

### 3.2. Intervention Outcomes

An overview of the data extraction of the records in terms of micro-level, meso-level, and macro-level results and factors influencing the effectiveness of the intervention are shown in Table 2. The included interventions are subdivided into twelve themes described in the following paragraphs.

#### 3.2.1. Onboarding

Multiple records have described that new nurses feel overwhelmed in the transition from student towards their new role as nurse [57,62,75,77], which suggests supporting those healthcare workers during this transition period can be beneficial. In addition, onboarding, the terminology used to describe new employees joining and integrating into the organization [85], is an important item.

Four of the included studies mainly focused on the onboarding program on the transition from nursing school towards the first job as a nurse and started at the last stage of nursing school [34,57,62,77]. First, Tseng et al. [77] studied an extensive externship program (EP), the Corporate-Academic Cooperation Program (CACP), to bridge the gap from nursing school to a clinical setting. During the CACP there was more focus on practicum arrangement, courses (e.g., career education and seminars), and establishing a collaborative partnership between the school and hospital. The control group received the standard EP. Students who participated in the CACP achieved a statistically significant improvement in retention rates relative to those who participated in the EP (*p* < 0.05) [77].

Furthermore, Kaihlanen et al. [62] studied the effect of the final clinical practicum (FCP) in Finland. The FCP focuses on student preparation for the upcoming transition to working life. FCP uses elements such as gaining learning experience mirroring the real work as graduated, being a professional team member, and receiving adequate support and supervisory relationships. They found a significant association between turnover intention and FCP (β = 0.21, *p* < 0.001).

To continue, Hernandez et al. [57] implemented the University of New Mexico College of Nursing (UNM CON)/University of New Mexico Hospital (UNMH) internship program in Mexico for new graduates. The focus of this internship program contained six items: focusing on organizing work and setting priorities, communicating effectively, developing clinical leadership skills, developing technical skills which are needed to provide safe care, practising quality care with actually sick patients, and learning to work in an emergency or end-of-life setting. A total of 43.3% of the participants who could have been employed for five years remained employed at the hospital after the internship program. In addition, 63.6% of the participants who remained employed at the hospital for five years or more continued to work in the exact location they had at the first year of their employment. There is no statistical test adjusted to study the retention rates.

Finally, The Academic Partnership Program (APU) of Aull et al. [34] included an evidence-based clinical education program designed to train, recruit, and retain Bachelor of Science students towards Bachelor of Science in Nursing (BSN) prepared nurses without the need for an academic faculty. The APU is a practice of students in a home-based department with a nursing preceptor serving as a clinical instructor. The students work with their instructor on multiple units as long as the program continues, which helps to integrate the student into the culture of their assigned unit. As a result, the turnover rate reduced from 23.9% nationally to 7% after the APU.

Six of the included studies focused on their onboarding program for new graduates [37,40,42,63,67,73]. Blegen et al. [37] studied the effect of a structured transition-to-practice (TTP) program for new graduates containing multiple online modules. The preceptor of the hospital needed to complete an online model for introduction to the TTP program, described the difference in high and low preceptor support, and the effect of this support program for new graduates. They found a difference in outcomes of new graduates getting high preceptor support (HPS) versus low preceptor support (LPS). The retention rates of HSP hospitals were higher (86%) at the end of the first-year program, whereas only 80% of the hired students at LPS hospitals were retained (*p* > 0.01) [37]. This shows that the intensity of preceptor support is an important factor in a mentorship program for onboarding new graduates [37].

In addition, Kang et al. [63] developed a situational initiation-training program (SITP). SITP focuses on the preceptor aiming to reduce stress levels and intention to leave of new graduates who have support from the preceptor. SITP contained four courses: “Covered preceptor roles, functions, and responsibilities; communication skills; stress management skills and relationship maintenance skills.” [63]. During the first preceptorship year, the new graduates showed low to shallow intentions to leave their current job at month three (mean = 4.18), six (mean = 3.8), nine (mean = 4.87), and twelve (mean = 2.6) [63].

Furthermore, Rudin et al. [73] studied the effect of the mentorship programme in Malaysia. The results showed a positive impact of the mentorship program on remaining in the nursing profession (r = 312, *p* = 0.001), though it is unclear how this mentorship program was set up in detail [73].

Koneri et al. [67] studied the one-year residency program with six touchpoints to focus on during the program. Touchpoints are defined by Koneri et al. [67] as: “distinct points in the company-customer experience.”. Whereas, employees are customer types of a company, Koneri et al. [67] designed a six-touchpoint program including: recognizing intrinsic worth (by sending personalized cards), developing loyalty (success stories featured in the newsletter), respect and dignity (monthly coffee-and-chat opportunities), valuing (organization of development day, educational events and sharing positive experiences) and trusting (inter-professionals teams focusing on communication, leadership, situation monitoring, and newly nurses’ support). The touchpoint program had a positive effect on retention rates compared to the non-intervention cohort (*p* < 0.00). The program had a cost-effective impact on retention (USD 180 versus USD 47,000) [67].

Additionally, Chen et al. [42] studied a three-month adaptive education program on learning, mental health, and work intentions. The education program led to an increase in the turnover rate of 12.6%, after three months of implementation, towards an 87.9% one-year retention rate. Unfortunately, no comparison is available for these turnover rates before implementation [42].

Lastly, Çamveren et al. [40] tested an organizational socialization model-based preceptorship program for nurses focusing on new graduates in transition. The preceptor must support the new graduate. The program contained preceptor training and support meetings for newcomer nurses. Both components contained feedback moments, which were used to improve the preceptorship program. At the end of the one-year program, there was no significant difference compared to the baseline in nurses’ intention to leave the unit or profession. Moreover, after the program, the results showed a significant increase in the nurses’ intention to leave their organization (t = −4.153, *p* < 0.001) compared to the year before. This study showed that not all preceptorship programs positively impact retention rates [40].

Three studies focused on a mentorship program regarding the onboarding [51,80,83]. Fleig et al. [51] described the effect of interpersonal mentoring as support for healthcare workers. Healthcare workers who received more interpersonal mentoring were more affectively committed to the organization (r^2^ = 0.35, F (3, 144) = 25.83, *p* < 0.01). This affective commitment moderated the effect of knowledge transfer and turnover intentions. Respondents who reported higher levels of knowledge transfer considered leaving the organization when their affective commitment was low. Though, knowledge transfer showed no significant direct relation with turnover intention (r^2^ = 0.09, F (3, 141) = 4.57, *p* < 0.01). The direct impact of the mentoring program on turnover intention was lacking [51].

Secondly, Williams et al. [80] focused on one-to-one mentorship, which was defined as “where a single mentor is assigned to a mentee”. The participants who received one-to-one mentoring rated the experience in helping transition to practice, professional development, and stress management higher than their colleagues. There was no significant relationship found between turnover intention and the two types of mentoring [80].

Last, Zhang et al. [83] investigated the one-to-one mentorship program for one year, where the mentee and mentor mainly focused on individual career development and the relationship, social support, and role modelling between both. They compared this mentorship program with a basic preceptorship program. For the one-to-one mentorship program, the mentor received an orientation program of four hours that focused on developing mentoring skills. The one-to-one mentorship program resulted in a significantly lower turnover rate (3.77%) in the first year than the control group (14.07%). The rates in the second and third years were not different [83].

Most of the above articles have affirmed the positive impact of onboarding programs on retention rates [37,42,51,57,62,67,73,77,80,83].

#### 3.2.2. Transition Program to a Different Unit

Three studies focused on the transition to a different unit [54,71,75]. Morphet et al. [71] studied the Transition to Specialty Practice Program (TSPP) for novice nurses entering a nursing specialty. TSPP offers a formal education and clinical support program combining “extended orientation, theoretical preparation, supernumerary time, preceptorship, and clinical support” [71]. Qualitative interviews indicated that the TSSP positively affected nursing recruitment in a studied emergency department. The organization and emergency ward became more attractive for the new nurses by focusing on education and support [71].

In addition, The Bridging Program of Gilroy et al. [54] focused on experienced paediatric nurses who wanted to develop and specialize in paediatric critical care. Gilroy et al. [54] did not execute the statistical analysis. However, they noticed that the external turnover rate of the participants of the program was 9%, which was lower than the overall unit turnover at that moment (12%) [54]. This outcome supports the positive outcomes of the other transition programs.

Finally, Schroyer et al. [75] focused on their Academy of Medical-Surgical Nurses (AMSN) Mentoring Program for nurses newly entering a specialty unit within critical care service, another transition during the career and stage of onboarding at another department and team. During the AMSN Mentoring Program, every newly entering nurse is paired with a mentor (experienced nurse) who provides guidance and nurturing. In the not-mentored group, 66% of nurses were retained, whereas 91% of the mentored nurses were retained (*p* = 0.001, chi^2^ = 6.873, 95% CI). Apart from that, nurses and trainees explained it was sometimes difficult to catch up with their mentors due to different shifts [75].

#### 3.2.3. Stress Coping

Healthcare workers are dealing with high-stress levels. Seven of the included studies revealed interventions focusing on coping skills to reduce the intention to leave [43,50,58,60,72,74,78]. Im et al. [60] set up a Huddling Programme in Korea for new nurses. The Huddling Programme contains four sessions within nine weeks of peer group activities focusing on empowerment. The programme focused on the mechanism of the group dynamic of nurses, which could help them cope with job stress and related problems [60]. Analyses revealed that turnover rates during the study period were lower for the intervention group (4.2%) than the control group (20.0%); however, they were not statistically tested [60].

El Khamali et al. [50] designed a five-day course for nurses. This course is intended to reduce job strain by improving the ability of ICU nurses to cope with stress by complementing medical knowledge and facilitating role-plays. The course led to significantly better numbers of retention than the control group (*p* = 0.04). The intervention costs the employer approximately EUR 2000 per nurse [50].

In the US, Hines et al. [58] implemented the American Nurses Association’s (ANAs) self-care guidelines in a small sample at the women’s and new-born service department. The ANAs guideline services tools to assist the nurses by selecting the appropriate self-care activities based on the particular stress in their workplace. The guideline resulted in a non-significant stress reduction (z = 0.58, *p* = 0.564) and a non-significant reduction in intent to leave (z = 1.13, *p* = 0.257) [58].

Additionally, Vardaman et al. [78] supported nurses with two computerized training sessions for Change-related Self-Efficacy (CSE). Self-efficacy is one’s belief in their ability to perform capably during any change [78]. Results showed that for every unit CSE increases, the turnover intention decrease by 0.46 (*p* < 0.01) [78].

Rushton et al. [74] set up the Mindful Ethical Practice and Resilience Academy (MEPRA), which enhanced a culture of mindfulness, ethical competence, and resilience. This was cultivated by six experimental workshops of four hours with six different elements, daily technology-enabled mindfulness and reflective practice, and reflective questions. MEPRA resulted in decreasing turnover intention (F = (3, 83), *p* = 0.05) [74].

Furthermore, Chu et al. [43] studied the use of nurses’ strength, which is defined as: “the characteristics of a person that allow them to perform well or at their personal best” [86]. Using nurses’ strengths was fulfilled by the included nurses using positive psychology and positive organizational behaviour [43]. They found out that nurses’ strengths use had a significant positive relationship with job crafting. Job crafting is defined as: “Nurses spontaneously changing the boundaries of cognition, tasks, and relationships of their work resulting in improving the job fit.” [43]. It seemed that strength use was significant positive related to job crafting (β = 0.68, *p* < 0.001). Job constructing was negatively correlated with turnover intention (β = −0.27, *p* < 0.01). This suggests that nurses’ strength use would decrease turnover intention, though this relationship was not significant (β = −0.01) [43].

In terms of self-care, a study conducted in the US studied the effect of four creative arts therapy programs [72]. The study aimed to allow healthcare workers to gain control over their psychological stress using visual art, musical practice, creative writing, or physical dance or movement. The intervention program showed improvements in turnover intentions (*p* = 0.001) [72].

#### 3.2.4. Social Support

Four of the included studies have examined social support, which may help with managing stress and possibly impact job retention [44,53,55]. Forde et al. [53] gave the nurses a moment to speak up by implementing and testing a ‘listening to staff’ event (L2S). After implementation, the turnover numbers decreased from 18.9% in October 2017 to 10.2% in October 2020.

Concilio et al. [44] exchanged a six-week digital intervention using text messaging. The text messages of the control group only contained medical facts in the experimental group. The text messages in the interventional group contained emotional, esteem, and networking support. The digital intervention derived an increasing sense of social support in the control group. Though, the intention to leave (BF = 2.459) did not change in the control or the experimental group [44].

Additionally, Guo et al. [55] had a valuable result with the WeChat Three Good Things That Happened (3GT). During this six-month intervention, the nurses were asked to record three good things that happened. Afterwards, they had to discuss why these good things happened and their role in making them happen. Using the WeChat 3GT intervention resulted in a significantly decreased turnover intention (F = 11.0323, *p* = 0.001) [55]. It should be noted that the WeChat 3GT intervention was tested on burnout nurses exclusively.

Lastly, Jensen et al. [61] set up a study to research the effect of facility dogs on healthcare workers. For this study, the participants had to work for at least six months with the facility dogs. The presence of facility dog had a significant association with turnover intention Healthcare workers who work with a facility dog reported reduced intentions to quit their jobs than the control group (β = −0.27, *p* = 0.002, d = −0.50) [61].

#### 3.2.5. Extra Staffing

With the shortage of healthcare workers, two studies revealed interventions contracting other personnel with a healthcare background (e.g., unlicensed assistive personnel) to support the nurse staffing and prevent them from leaving [49,81]. Winslow et al. [81] constructed a partnership Care Delivery Model (CDM) in a Magnet hospital in the US. The project was designed using a dyad or triad comprised of two nurses, one nurse and one unlicensed assistive personnel, two nurses, and one unlicensed assistive personnel taking care of a team of patients. The partnership CDM did not result in significant differences in the nurse turnover [81].

Furthermore, at an Acute Care department in Australia, Duffield et al. [49] added unregulated nurses support workers (unlicensed) to existing nurse staffing. Wards where nurse support was added, had non-significant higher numbers of nurse intent to leave [49].

#### 3.2.6. Coping with the Demands of Patient Care

The primary responsibility of healthcare workers is patient care. Two included studies revealed tools which may support novice nurses in coping with the demands of patient care [76,84].

Zhong et al. [84] tested the humanistic care teaching model. Paediatric nurses practised this patient-care model by writing various clinical cases and practising with organized role-plays. It showed that the turnover intention in the intervention group was significantly lower than in the control group (*p* < 0.001) [84].

Tang et al. [76] suggested that novice nurses have a hard time prioritizing and managing the health problems of their patients. The Humanoid Diagram Teaching Strategy (HDTS) was implemented to help novice nurses reintegrate their knowledge and skills to make decisions. The training started after the first month of pre-service training and was conducted three times a week for four weeks consecutively. During this training, the patient’s appearance was drawn in three parts: the head and neck, trunk, and limbs. The clinical preceptor encourages the novice nurse to employ association thinking and use guidance and discussion. The goal of the HDTS was to identify the primary patient problems and solutions, which resulted in learning how to manage specific cases. This HDTS resulted in a significant difference in the retention rate of the intervention group (β = −0.33, *p* < 0.005) [76].

#### 3.2.7. Work Relationships

Work relationships have an impact on the retention rates of healthcare workers, which was shown in five of the included studies [31,35,56,64,65]. In 2017, Kang et al. [65] published the effect of the cognitive rehearsal program for nurses on interpersonal relationships with ten topics: nonviolent communication, withholding information, backbiting, sabotage, disgracing, undermining activities, failure to respect privacy, physical aggression, verbal affront, and self-empathy. After the intervention, the intervention and comparison groups showed significant differences in intention (F = 5.55, *p* = 0.024), which continued up to four weeks after the intervention program [65].

Secondly, Harris et al. [56] studied the effect of communication strategies. Specifically, the communication strategy contained clinician training using situation, background, assessment, and recommendation (SBAR) twice daily shift huddles with the BAR method and a monthly clinician meeting over three months. The communication strategies led to a decrement in unit turnover from 7.84% to 2.33% at the end of the three-month project. The increased cost for this project occurred with staff meetings being held once a month. Half of these individuals were paid an extra hour for attending this meeting, and the other half were already present for their shifts [56].

Then, Baik et al. [35] set up five four-hour interprofessional team training: a team intervention including team strategies and tools to enhance performance and patient safety (TeamSTEPPS) and communication training. Furthermore, the team followed quarterly leadership workshops. Lastly, a structured bedside rounding was implemented. The six months turnover rate before the interventions was 5.74%. Post-intervention, this turnover rate decreased to 5.3%. The retention rates were not statistically tested [35].

Kang et al. [64] developed a smartphone application to cognitively train nurses to cope with bullying situations in the workplace. The application consists of an introduction to nonviolent communication as the standard, six digital comic drawings of workplace bullying situations and nonviolent communication strategies, and a board for questions and answers. The intervention effectively decreased nurses’ person-related bullying and the experiences of work-related bullying. Pre-measurement the mean (SD) was 3.26 (0.81). The smartphone application decreased retention rates at four-week implementation by 3.13 eight-week measurement 3.36 (0.77). Whereas, the mean (SD) score of the control group was 3.59 (0.84), 3.66 (0.84), and 3.67 (0.71), respectively [64].

Lastly, Al Sabei et al. [31]) researched the impact of interprofessional teamwork. This practice is characterized by shared team identity, clarity, shared responsibility, integration, and independence, on the intention to leave of nurses. Interprofessional teamwork was directly associated with nurses’ intention to leave and indirectly mediated by job satisfaction and burnout [31]. The study did not reveal how the interprofessional teamwork intervention was precisely examined in practice [31].

#### 3.2.8. Development Opportunities and Department Resources

Opportunities in the development of the workforce and resources may help through the retention of nurses and physicians. Three included studies discussed certain interventions [46,47,69]. In Australia, Dawson et al. [47] studied supportive strategies. The strategies contained providing employment options, rewarding performance, enhancing professional development and training, and improving management practice [47]. However, Dawson et al. [47] did not describe concrete results of these strategies.

Furthermore, Dawood et al. [46] set up a qualitative study to discuss the effect of dual roles: working as a nurse and an emergency nurse practitioner (ENP), as an intervention to improve retention. If the dual role was not available, most part-time ENPs did not consider leaving nursing altogether. However, full-time participants without dual roles considered leaving nursing, confirming that dual roles could force retention [46].

Moreover, Melnyk et al. [69] focused on the idea that implementing evidence-based practice (EBP) will result in renewing the nurses’ professional spirit and giving them a voice [87,88] which may have a positive impact on job satisfaction [87]. Melnyk et al. [69] used the Advancing Research and Clinical Practice through Close Collaboration (ARCC) model to implement EBP. EBP culture and EBP mentorship resulted in being key variables that significantly positively impact the intention to stay among nurses (*p* = 0.02) [69].

Brabson et al. [38] focused on the three EBP training models for physicians: “Cascading model, learning collaborative, and distance education.” [38]. Results showed no differences in turnover rates at the 12-month measurement point (χ^2^ (2, *n* = 96) = 2.10, *p* = 0.35, Cramer’s V = 0.15) or at the end of the study (χ^2^ (2, *n* = 95) = 0.51, *p* = 0.77, Cramer’s V = 0.07) [38].

#### 3.2.9. Job Environment

Six studies demonstrated interventions by influencing the job-/work environment to impact the retention rates [30,32,39,48,70,79]. Brewer et al. [39] studied the effect of a Magnet hospital. The Magnet Recognition Program^®^ acknowledges healthcare institutions that offer exceptional nursing care and working environments through an inventive program. It seemed that working in a Magnet hospital did not significantly impact turnover intentions (coefficient (CI) = 0.039 (−0.150 to 0.227), *p* = 0.687) [39].

Mohamadzadeh et al. [70] compared the outcomes of excellence-awarded hospitals to the outcomes of hospitals that do not have an excellence plan. The European Foundation for Quality Management (EFQM) is an excellent plan which has three levels. At the first level, eight criteria have been considered to evaluate the performing hospitals. These eight criteria were: leadership, policy and strategy, employees (human resources), participations and resources, customers’ results, employees’ results, and societies and performance key results. At the second level, the criteria were described in detail using subsets. At the third level, a list of specific guidelines regarding more explanation of each subset was available. The score means of intention to leave the organization in performing and non-performing organizations of the excellence plan showed a significant difference (*p* = 0.004). Performing the organizational excellence plan reduced the intention to leave [70].

In terms of environment, Alvaro et al. [32] tested the impact of the architectural design of the hospital on patient and staff outcomes using a pretest-posttest quasi-experimental study. The new design mainly focused on creating an architecture of wellness containing communal dining spaces on each floor, public spaces, multiple outdoor terraces, and a rooftop terrace with views of the skyline, lake, and green environment. Workplace satisfaction of healthcare workers did improve (*p* = 0.000). There was no significant difference in intention to quit staff. Though, staff with favourable impressions of the new architectural design and a greater sense of belonging to the neighbourhood showed a decreased intention to quit (*p* < 0.01) [32].

Furthermore, Walker et al. [79] studied the effect of the integration of an electronic health record on retention rates. Quality of care did improve significantly in terms of infections, pressure ulcers, and falls (*p* < 0.01). Though, the analysis of data revealed no significant model effect (F (2, 42) = 2.09, *p* > 0.05, r^2^ = 0.07), nor did the model explain the variance in the nurse turnover [79].

Adams et al. [30] explored the impact of the Cultural Change Toolkit on the nursing work environment. The toolkit provides information and tools that encourage positive practice changes. It mainly focuses on meaningful recognition, shared decision-making, and increasing leadership support and involvement. The implementation of the toolkit led to a reduction in the anticipated turnover scale (mean rate pre-implementation = 3.133, post-implementation 2.989), though this reduction was not significant [30].

Finally, Deng et al. [48] studied the comprehensive reform in a hospital in China. The government implemented new policies on personnel, compensation, management, and diagnosis and treatment. Details can be found on the website of the Beijing Municipal Health Commission Information Centre [89]. Four years after implementation, the average annual growth rate was 9.1% for nurses and physicians in Beijing public hospitals. The turnover intention thought of 61.4% had decreased [48].

#### 3.2.10. Work Organization

Four included records studied the impact of the work organization on retention rates [36,45,68].

Daniels et al. [45] studied the effect of the ‘70% Full-Time Commitment’. A provincial government in Ontario, Canada, developed this strategy where at least 70% of the nurses work full-time, and the other 30% work part-time or casually. It aimed to stimulate working full-time. Results showed that the ‘70% Full-Time Commitment’ seemed to be no effective intervention in retaining part-time and casual nurses [45].

In a qualitative study in the UK by Baillie et al. [36] nurses changed from twelve-hour to eight-hour day shifts. It appeared that the eight-hour day shifts negatively affected recruitment and retention, mainly because an increased amount of staff members were needed to cover the eight-hour day shift pattern [36].

Two selected studies checked the effect of the self-scheduling [68,82]. It was suggested that self-scheduling created a better work-life balance [68] and ensured more flexibility [82], possibly resulting in decreased turnover rates. Kullberg et al. [68] compared fixed scheduling with self-scheduling. Self-scheduling was significantly associated with more requests from management for short notice shift changes, whereas fixed scheduling was associated with less overtime. Self-scheduling showed overall relatively low to moderate levels of staff turnover compared to the fixed scheduling [68]. No significant calculations were executed.

In the US, a study with a larger group of nurses (*n* = 1497) in four hospitals was conducted by Wright et al. [82] to study the effect of self-scheduling. Two hospitals showed an absolute increase in turnover rates (1.5% and 1.4%), and two other hospitals reported an absolute decrease in turnover rates (−5.3% and −5.4%) [82]. The isolated effect of self-scheduling on retention rates was not described by Wright et al. [82] because no other variables were not studied.

#### 3.2.11. Recruitment Approach

Three studies concentrated on the recruitment approach as an intervention to retain nurses and physicians [33,52,66]. Two included studies focused specifically on the recruitment of physicians [33,52]. Firstly, Fleming et al. [52] studied the effect of provisional licensing to attract international medical graduated physicians who are without the licensing unable to work in Canada. The study showed international medical graduates started practice as a result of the provisional licensing but did not result in long-term retention [52].

Secondly, Arora et al. [33] set up a special rural recruitment track for physicians in the rural area of Thailand. In Thailand, the Collaborative Project to Increase Production of Rural Doctors (CPIRD) and the One District One Doctor (ODOD) project, were set up to increase the number of doctors in rural areas. Arora et al. [33] studied the long-term effect of these two recruitment projects. It seemed that doctor retention was higher in areas where the initiatives were implemented than in the regular tracks (*p* < 0.05) and medical were 2.4-fold more likely to remain working for the area for a minimum period of three years (OR (95% CI) = 2.44 (2.19–2.72)) [33].

Finally, the Prospective Staffing Model researched by Kester et al. [66] focused on the implementation of a model to predict preventable and potential turnover at a thoracic surgery department. Restructure of the recruitment strategy was included in the implementation of the prediction model. It involved engaging current workers in the interview process and prioritizing the candidates regarding desirable characteristics. Furthermore, an internal nurse recruiter organized interviews and had weekly meetings with the nurse manager to improve the partnership. The hospital empowered local academic partners such as colleges and universities to improve the knowledge about the new graduates. Additionally, the length of the orientation of the newly hired nurses was enlarged towards eight weeks for experienced nurses and towards 12 weeks for new graduates. The implementation of the prediction model led to a 17.6% decrease in turnover in a four-year period. The cost of the 12-week orientation was $11,066.40 in 2018, which is still less than the average cost for the replacement of a new employer (about $52,100) [66].

#### 3.2.12. Technological Innovations

There are technological healthcare interventions innovated to increase retention, two of the included records focused on the use of robots in healthcare [41,59]. Chang et al. [41] set up robots to help nurses focus on professional task engagement. They found that robot-enabled focus (“nurses’ perception that robots enable nurses to concentrate on conducting major nursing jobs” [41]) on professional task engagement positively impacted overall job satisfaction (r = 0.31, *p* < 0.05) and perceived health improvement (r = 0.34, *p* < 0.05). Robot-reduced nonprofessional task engagement (“nurses’ perception that robots help share the workload of auxiliary jobs” [41]) was positively related to only perceived health improvement (r = 0.26, *p* < 0.05). Furthermore, Chang et al. [41] noticed that job satisfaction and perceived health improvement were negatively related to turnover intention (r = −0.41, *p* < 0.05 and r = −0.18, *p* < 0.05) [41]. These findings suggest that, by using robots, the increased focus of nurses on professional task engagement and reduced focus on nonprofessional task engagement could help to improve job satisfaction and job retention of nurses [41].

Huang et al. [59] tested the effect of effort ensuring smooth operation (EERSO), “the time and energy needed to keep robots operating as designed”. EERSO was positively associated with time pressure (β = 0.16, *p* = 0.007) and missed care (β = 0.13, *p* = 0.003). Using robots may help reduce nurses’ workload by focusing on nurses’ saved time and, therefore, turnover intention. However, it also requires nurses’ efforts to maintain EERSO, which may adversely impact nursing professional workplaces [59].

## 4. Discussion

This systematic review resulted in an overview of the existing interventions for job retention of nurses and physicians in a hospital setting. The included records resulted in twelve themes on which management could focus on in terms of job retention: onboarding, transition program to a different unit, stress coping, social support, extra staffing, coping with the demands of patient care, work relationships, development opportunities and department resources, job environment, work organization, recruitment approach, and technological innovations.

The positive impact of the onboarding program [34,37,42,57,62,63,67,77] and mentorship [51,73,80,90] is in line with earlier published systematic reviews [91,92]. Kakyo et al. [93] explored the benefits of the informal mentoring program for nurses and confirmed that built on the reciprocal relationship between mentee and mentor; there is a substantial benefit of the mentoring program.

Furthermore, the onboarding program shows the importance of supporting the new graduates within the first two years of their working life [37,42,63,67]. More than 50% of newly graduated nurses leave their job within the first year due to culture shock [94]. To prevent them from leaving early in their working life and negatively impacting the staff long-term, it seems important to focus on and maintain this specific group. Stevanin et al. [95] described the difference in stress reporting between generations (e.g., baby boomers, generation x, and generation y). It showed that generation y reports more psychological stress than previous generations and requires support in their workplace [95]. It is suggestible that new generations (generation y and subsequent) have substantial needs to support them in the overwhelming transition toward their new role [57,62,75,77], than their previous colleagues. It makes the importance of onboarding programs, focusing on new graduates and new generations starting their careers and dealing with stress due to the transformation from students towards registered nurses, even more clear.

In this systematic review, none of the included records studied onboarding programs for physicians. It illustrates this content is missing in research and makes it questionable if physicians could profit from an onboarding program. A systematic review published in 2021 affirmed the relevance of early clinical contact during medical school and the early postgraduate period for the retention of physicians in a rural setting [96]. Additionally, Kumar et al. [96] also underscored the impact of professional and personal support on the retention rates for this group. Hence, onboarding programs, that focus on early clinical contact and support, could be beneficial for physicians, same as for nurses.

In addition, this systematic review highlighted the importance of tools for stress coping [43,50,72,74], though all of them focused specifically on nurses. It seems reasonable that physicians are dealing with stressful situations, likely as nurses. Unfortunately, interventions focusing on physicians coping with these stressful situations are lacking in this review. A review by Darbyshire et al. [97] confirmed that physicians in an acute care setting have a need for stress management techniques. These techniques could positively impact retention rates [97]. These findings make it highly likely that, for example, copings tools for stress management or mentorship programs could also be effective for physicians.

Interestingly, the interventions included in this systematic review do not mention salary as a solution for upgrading the retention rates for nurses and physicians. Earlier research showed that the migration of healthcare workers is, among other things, caused by the lower salaries in low- or middle-income countries [5,6]. A literature review by Okafor et al. [98] explained that the migration of nurses in Nigeria is affected by the worse payment and pushed nurses towards countries with better working conditions and better pay. Due to the withdrawal effects of healthcare workers’ migration to low- or middle-income countries, higher salaries may help reduce the intention to leave and migration [5,6,98], though it may not be the most cost-effective intervention [99]. An earlier systematic review revealed that salary is not the most common reason nurses and physicians leave their jobs in high-income countries; job satisfaction, work-life balance and social support are frequently named determinants that impact the intention to leave [25]. This suggests that salary impact may vary per low-, middle-, or high-income country. It is suggestible that salary is not a primary reason for leaving healthcare in high-income countries. Nevertheless, it is an important basis from wherefore leaving and thus a vital basis managers can build to rice retention. Managers must implement specific retention interventions that match the determinants that apply to the concerning culture or country.

However, implementing cost-effective retention interventions must likely overcome some barriers before success. For example, structural barriers such as staff workload and lack of time are commonly described as barriers to the implementation of hospital-based interventions [100] To overcome these barriers, it seems essential to enhance commitment and motivation of the staff by convincing them of the advantages for the staff themselves and sharing success stories [100].

Although a great effort was made to create a funded systematic review, there were some limitations. Firstly, a meta-analysis is not conducted due to the heterogeneity of the included records. Secondly, the authors may have missed some studies as a result of the exclusion of grey literature. The grey literature was excluded because the extensive search led to a large number of results and a comprehensive results paragraph. Lastly, it is feasible that the chosen themes of interventions overlap, which can create bias. This overlap demonstrates that the interventions affect multiple determinants that could positively impact retention rates. To maintain the retention intervention impacts all possible determinants, it is crucial to implement it on various organizational levels.

This systematic review studied extant literature on both physicians and nurses, which constitutes a key strength of this study. Though, it stands out that limited interventions that were included in this systematic review contained interventions for physicians. Other systematic reviews of this topic focused on early careers or only experienced nurses [91,92]. To the best of our knowledge, there is no systematic review available exploring interventions for improving retention in a hospital setting for both nurses and physicians, which makes this systematic review unique. Nevertheless, a higher number of the included records included nurses instead of physicians. Applying the results of this study to physicians in a hospital setting can create bias due to a lack of research concerning physicians. Accordingly, the outcomes should be handled with care implemented for physicians. Nonetheless, numbers showed that the shortage of physicians leaving healthcare is just as alarming as the nursing rates [1,2]. The literature describes high numbers of physicians dissatisfied with their jobs and burnout symptoms related to higher turnover rates [7]. A possible explanation could be that researching the intention to leave is taboo in medical culture among physicians. This might result in a minimal to not accessible target for research in retention interventions or implementation of retention strategies. Hence, the authors of this review suggest focusing on enlarging the importance of researching physicians’ intentions to leave.

Moreover, this review has thus far focused on one-factor interventions that impact the intention to leave or stay. However, job retention showed inter-correlation with other determinants (such as job satisfaction, burnout symptoms, job demands and job resources) that could also be impacted using interventions [28]. This effect is minimally studied. Hence, research on this topic could help to adjust the impact on a broader level.

Lastly, the transition from school to work seemed a vital deal breaker for nurses [57,62,75,77]. This raised the question about the extent to which nursing school actually prepares students for the skills they need to start work as a nurse. More research is undoubtedly desirable to prevent new graduates from leaving their workforce.

## 5. Conclusions

The outflow of nurses and physicians leaving hospitals is enormous. The impact of COVID-19 increases the urgency in preventing nurses and physicians from leaving. This systematic review resulted in multiple interventions that can be used to upgrade retention rates. Additionally, the implementation of organizational change and the establishment of mentorship programs are important interventions.

When selecting an intervention for implementation, managers and human resources should focus on the characteristic intervention that matches their healthcare workers and the hospital’s mission, vision, and values statements. Sharing the success stories of implanted interventions may be advantageous for all healthcare organizations.

In summary, this review can contribute to implementing retention interventions in hospitals, which can aid in maximizing retention, especially for nurses. Furthermore, this review can contribute to planning future studies containing more physician-specific interventions.

## Figures and Tables

**Table 2:** Data-extraction table micro, meso, macro results and factors influencing the effectiveness of the intervention.

First Author (Year)	Micro Results (Individual)	Meso Results (Department)	Macro Results (Hospital and Further)	Factors Influencing the Effectiveness
Adams, A. (2019) [30]	Reduction in burnout scores (mean burnout score, pre-implementation = 4.808, post-implementation = 4.463, *p* = 0.004).	A reduction in the overall mean rate of turnover based on the anticipated turnover scale results, but no statistically significant change.	Unknown	Unknown
Al Sabei, S.D. (2022) [31]	Unknown	Interprofessional teamwork is directly associated with the intention to leave.	Unknown	Job satisfaction and job burnout indirectly mediate the influence of teams. work on the intention to leave
Alvaro, C. (2016) [32]	General well-being of staff did not improve. Optimism, burnout of staff no difference. Workplace satisfaction (*p* = 0.000) and workplace interaction (*p* = 0.000) did improve	Intention to quit did not change after intervention.	Unknown	Staff with favourable impressions of the building design and a greater sense of belonging to the neighbourhood demonstrated decreased intention to quit (*p* < 0.01).
Arora, R. (2017) [33]	Unknown	Retention was significantly higher in those hospitals under special recruitment (*p* < 0.05). Medical graduates under the special rural recruitment scheme were more as two-fold more likely to remain for a minimum period of three years (OR (CI) 2.44 (2.19–2.72)).	Unknown	Unknown
Aull, M. (2022) [34]	Unknown	Reduction of turnover (7% instead 23.9% national)	Unknown	Unknown
Baik, D. (2019) [35]	Higher scores of satisfaction with their job after intervention (Mean (SD) = 4.46 (0.74), *p* = 0.001) than before (Mean (SD) = 3.95 (0.51).	The six-month period turnover rate reduced from 5.74% pre-intervention into 5.3% post-intervention.	Unknown	Unknown
Baillie, L (2019) [36]	Unknown	Negatively affect recruitment and retention.	Unknown	Unknown
Blegen, M.A. (2015) [37]	Nurses in HPS were rated high for quality of improvement, EBP, technology, and teamwork and communication than their colleagues in LPS hospitals (*p* < 0.05).	At the end of the first year, 86% of the nurses by HSP hospitals whereas by LSP hospitals only 80% retained (*p* < 0.01).	Unknown	Unknown
Brabson, L.A. (2019) [38]	Unknown	There were no significant differences in the rates of turnover for clinicians in each training condition at the 12-month time point or by the end of the study.	Unknown	Unknown
Brewer, C.S. (2012) [39]	Unknown	No significant difference in turnover intention (coefficient (CI) = 0.039 (−0.150 to 0.227), *p* = 0.687) in working in a Magnet hospital.	Unknown	Unknown
Çamveren, H. (2022) [40]	Significant decrease in nurses affective organization commitment (t = 4.443, *p* > 0.001), their normative organizational commitment (t = 3.433, *p* < 0.001), and professional affective commitment (t = 7.390, *p* < 0.001) after one year of preceptor program.	A significant increase in the newcomer nurses’ intention to leave their organization (t = −4.153, *p* < 0.001) and no difference in intention to leave the unit or profession (*p* > 0.05).	Unknown	Unknown
Chang, H.Y. (2021) [41]	Robot-enabled focus on professional task engagement was positively associated with job satisfaction and perceived health improvement. Robot-reduced nonprofessional task engagement was positively related to perceived health improvement.	Unknown	Unknown	Overall job satisfaction and perceived health improvement were negatively related to turnover intention.
Chen, S. (2021) [42]	Increase of self-care, an increase of care of learning.	After the intervention of the overall plan, the turnover rate of new graduate nurses within three months after implementation the turnover rate was 12.6%. One year after the overall plan the rate was 87.9%	Unknown	The positive outcomes of the intervention are related to the instructor’s care.
Chu, X. (2022) [43]	Unknown	Strength use had a significant positive relationship with job constructing. Job crafting was negatively correlated with turnover intention (β = −0.27, *p* < 0.01). No significant relationship was found between nurses’ strength use and turnover intention (β = −0.01)	Unknown	
Concilio, L. (2021) [44]	The medical facts in the digital intervention increased the sense of social support.	Intention to leave the jobs, intention to leave the organization, and intention to leave the profession (BF = 2.459).	Unknown	Unknown
Daniels, F. (2012) [45]	Unknown	The intervention was not effective in retaining part-time and casual nurses.	Unknown	Unknown
Dawood, M. (2019) [46]	Unknown	If the dual role were not available, most part-time ENPs did not consider leaving nursing altogether. However, full-time participants without dual roles considered leaving nursing, confirming that dual roles could force retention.	Unknown	Inspiring aspects such as ‘great opportunity to develop clinical skills’ and ‘direct patient contact’, should be considered in creating new duo roles.
Dawson, A.J. (2014) [47]	Unknown	Unknown	Unknown	Nursing turnover is influenced by the experiences of nurses. Strategies that nurse managers could do to improve retention are improving performance management and work design.
Deng, J. (2019) [48]	After the pilot, 40.9% of the participants thought their health had improved (40.9%), challenge (37.5%) and hindrance stress (48.25) had decreased, public service motivation had increased (17.7%), job satisfaction had increased (54.4%), presentism had decreased (37.2%), their job performance had increased (61.1%), and quality of healthcare had improved (56.3%).	After the pilot, the number of healthcare workers in hospitals increased from 140,304 in 2011 to 198,290 in 2015, an average annual growth rate of 9.1%. Of the participants 61.4% thought their intention to leave had decreased.	Unknown	Unknown
Duffield, C. (2018) [49]	On nurse support wards higher quality of care (96.6%) was reported compared to regular wards (82.1%).	No significant different in intention to leave on nurse support wards comparing to regular wards in terms.	Unknown	Unknown
El Khamali, R. (2018) [50]	Absenteeism during follow-up period was 1% in the intervention group and 8% in the control group (between-group difference, 7% [95% CI, 1–15%]; *p* = 0.03). The prevalence of job strain at follow up period was 13% in the intervention group and 67% in the control group (between-group difference, 54% [95% CI, 40–64%]; *p* < 0.001).	The prevalence of leaving the ICU was lower in the intervention group compared with the control group (respectively, 4% versus 12%; between-group difference, 8% [95% CI, 0–17%]; *p* = 0.04).	Unknown	Unknown
Fleig-Palmer, M. (2015) [51]	More interpersonal mentoring results into more affectively committed healthcare personnel (r^2^ = 0.35, F (3, 144) = 25.83, *p* < 0.01).	More learning on the job were not more likely to leave the health care organization (r^2^ = 0.09, F (3, 141) = 4.57, *p* < 0.01) as there was an inverse relationship between knowledge transfer and retention.	Unknown	The relationship between knowledge transfer and turnover intention was moderated by affective commitment.
Fleming, P. (2012) [52]	Unknown	The intervention leads to an increase in medical graduates but does not lead to long-term retention.	Unknown	Unknown
Forde-Johnston, C. (2022) [53]	Unknown	Nursing turnover decreased from 18.9% to 10.2% after implementation.	Unknown	Unknown
Gilroy, H. (2020) [54]	Unknown	The turnover rate for participants is lower than the overall unit turnover (respectively, 9% vs. 12%).	Unknown	Unknown
Guo, Y.F. (2020) [55]	Led to a decrease in negative coping style (F = 6.020, *p* = 0.017) and improvement in positive coping style (F = 9.45, *p* = 0.003).	Significantly decrease turnover Intention (F = 11.0323, *p* = 0.001)	Unknown	Unknown
Harris, K.K. (2017) [56]	Unknown	Unit turnover decreased at baseline to the end of the three-month project (respectively, 7.84% vs. 2.33%)	Increasement of patient experience.	Unknown
Hernandez, S.H.A. (2020) [57]	Unknown	Of the healthcare workers who could have been employed for five years, 43.3% remained employed at the hospital. For those who remained employed at the hospital for five or more years, 63.6% continued to work in the same location as they had at the first year of employment	Unknown	Unknown
Hines, M. (2019) [58]	Not significant in stress reduction post intervention (z = 0.58, *p* = 0.564).	Post-intervention, a not significant reduction of intent to leave the organization was found (z = 1.13, *p* = 0.257)	Unknown	Unknown
Huang, T.L. (2022) [59]	Unknown	EERSO was positively associated with time pressure (β = 0.16, *p* = 0.007) and missed care (β = 0.13, *p* = 0.003). Using robots may help reduce nurses’ workload by focusing on nurses’ saved time and, therefore, turnover intention workplaces.	Unknown	
Im, S.B. (2016) [60]	The mean scores for normative commitment and impact of empowerment were higher in the experimental group, but ego-resilience did not differ significantly between the two groups (F = 5.106, *p* = 0.029 and F = 6.781, *p* = 0.012).	The percentage of staff turnover in the experimental group was 4.2%, whereas 20% in the control group.	Unknown	Unknown
Jensen, C.L. (2021) [61]	Working with a facility dog showed a significant association with personal accomplishment (β = 0.42, *p* < 0.001, d = 0.91) and greater positive affect (β = 0.29, *p* < 0.001, d = 0.62). Furthermore, working with a facility dog was also a significant predictor of less negative affect (β = −0.18, *p* = 0.031, d = −0.30), of less depression (β = −0.20, *p* = 0.025, d = −0.40), better overall mental health (β = −0.21, *p* = 0.017, d = −0.47), of better perceptions about the job overall (β = 0.25, *p* = 0.004, d = 0.57), of greater job-related enthusiasm and less job-related depression (β = 0.24, *p* = 0.005, d = 0.48), better affect balance (β = 0.27, *p* = 0.001, d = 0.53).	Results showed a significant association between facility dog presence and turnover intention (β = −0.27, *p* = 0.002, d = −0.50).	Unknown	Unknown
Kaihlanen, A.M. (2020) [62]	Unknown	The intervention was statistically significantly associated with turnover intentions.	Unknown	The effect on turnover intention is mediated by psychological distress, role conflict and ambiguity.
Kang, C.M. (2016) [63]	Unknown	During the first preceptorship year, the participant reported low intention to leave their current jobs at months 3, 6, 9, and 12 (mean = 4.18, 3.8, 4.87, and 2.6, respectively)	Unknown	Unknown
Kang, J. (2019) [64]	Unknown	The mean (SD) scores of turnover intentions at premeasurement, four-week measurement, and eight-week measurement in the intervention group were 3.56 (0.81), 3.13 (0.92), and 3.36 (0.77), and 3.59 (0.84), 3.66 (0.84), and 3.67 (0.71) in control group. The rehearsal intervention was effective in decreasing nurses’ person-related bullying and work-related bullying experiences.	We analysed the differences between the ICU and the general unit within each group to determine the effect of the type of unit. There were no significant differences between the ICU and the general unit in intention to leave.	Unknown
Kang, J. (2017) [65]	After the intervention, there were significant differences in interpersonal relationships between the experimental and control group (F = 6.21, *p* = 0.022).	The study showed significant differences in turnover intention (F = 5.55, *p* = *0*.024) between the intervention and control group.	Unknown	Unknown
Kester, K.M. (2020) [66]	Unknown	Implementation of the intervention led to a decrease in turnover of 17.6% in a four-year period.	Unknown	Unknown
Koneri, L. (2021) [67]	Post-intervention job satisfaction score was significantly higher (*p* = 0.05) than the pre-intervention.	The retention rate was significantly higher in the intervention group compared to the control group (*p* = 0.000).	The intervention had a positive and cost-effective impact on retention rates.	Job satisfaction
Kullberg, A. (2016) [68]	No differences in short-term sick leave between wards with fixed or self-scheduling	Self-scheduling showed relatively low levels of sick leave and low to moderate levels of staff turnover compared to fixed- scheduling. Self-scheduling was associated with more requests of short-notice shift changes. Fixed scheduling was associated with less overtime and fewer possibilities to change shifts compared to fixed-scheduling. Statistically significant differences in the safety of inpatient care (*p* = 0.0298).	Unknown	Unknown
Melnyk, B.M. (2021) [69]		EBP culture and EBP mentorship positively impacted intent to stay among nurses (*p* = 0.02).		
Mohamadzadeh Nojehdehi, M. (2015) [70]	Unknown	Performing the organizational excellence plan reduced the intention to leave the organization (*p* = 0.004).	Unknown	Results showed an inverse association between organizational climate and the intention to leave (*p* = 0.001)
Morphet, J. (2015) [71]	Participants showed an improved skill mix.	Nursing retention improved.	The intervention was reported to make the organization more attractive, by promoting focussing on education and support. The interventions had a positive effect on nursing recruitment.	Unknown
Moss, M. (2022) [72]	The intervention had improvements in anxiety- depression- total posttraumatic stress disorder, and burnout scores (*p* < 0.001).	Improvement of turnover intention (*p* = 0.001).	Unknown	Unknown
Rudin, N.M.N. (2018) [73]	Unknown	Mentored nurses were significantly more likely willing to stay in the nursing profession (r = 0.61, *p* < 0.01).	Nurses feel positive about nursing in their current hospitals (r = 0.75, *p* < 0.01 and are committed to professional nursing standards (r = 0.48, *p* < 0.05).	Unknown
Rushton, C.H. (2021) [74]	After implementation of the intervention ethical confidence (F = 73.27, *p* < 0.001), ethical competence (F = 29.32, *p* < 0.001), resilience (F = 18.2, *p* < 0.001), work engagement (F = 17.53, *p* < 0.001), and mindful awareness and attention (F = 4.78, *p* = 0.03) increased significantly. Furthermore, symptoms of depression (F = 5.78, *p* = 0.02) and anger (F = 5.82, *p* = 0.02) of the participant had reduced.	Turnover intentions decreased after the intervention (F = 3.83, *p* = 0.05)	Unknown	The intervention was more effective at decreasing emotional exhaustion for nurses in non-ICU wards than for those in ICU wards (*p* = 0.04).
Schroyer, C.C. (2020) [75]	Unknown	A higher percentage of mentored nurses retained compared to not-mentored nurses (91% vs. 66%), (*p* = 0.001, chi2 = 6.873, 95% CI).	Unknown	Participants found it hard to catch up outside work due to working in different shifts).
Tang, Y. (2022) [76]	Unknown	Between the intervention and control group, the retention rate was significantly different during two measurement moments after implementation (B = −0.33, *p* < 0.005).	Unknown	Test is performed by novice nurses
Tseng, C.N. (2013) [77]	Students who participated in the program had a statistically significant improvement in nursing competence (*p* < 0.01).	Participants in the cooperation program achieved a statistically significant higher retention rates *p* < 0.05).	Unknown	Unknown
Vardaman, J.M. (2020) [78]	For every one-unit increase in job embeddedness, self-efficacy is increased by 0.42 (*p* < 0.01).	For every one-unit increase in self-efficacy, turnover intention goes down by 0.46 (*p* < 0.01).	Unknown	Results show that self-efficacy manifests the effect of job embeddedness on turnover intentions.
Walker-Czyz, A. (2016) [79]	Unknown	There was no significant effect model of turnover data.	Unknown	Unknown
Williams, F.S. (2018) [80]	Individuals who received one-to-one mentoring rated the experience higher in helping transition to practice, professional development, and stress management.	No significant relationship between the type of mentoring and turnover intention.	Unknown	Nurses with a high degree of discomfort as a nurse were significantly more similar to a higher score of intention to leave (χ^2^ (2) = 24.91, *p* ≤ 0.001). There was a significant relationship between low frequency group mentoring and turnover intent (χ^2^ (1, *n* = 138) = 3.85, *p* < 0.05.
Winslow, S. (2019) [81]	No significant result.	No significant results.	Unknown	Unknown
Wright, C. (2017) [82]	Unknown	RN turnover decreased at two of the participating hospitals and increased at the other two participating hospitals.	Unknown	Unknown
Zhang, Y. (2019) [83]	Unknown	The findings showed that the turnover rates for the experimental group were at the end of the first (3.77%), second (3.48%), and third year (8.11%) as compared to 14.07%, 9.36%, and 14.19% for the control group. The survival curves were significantly different (*p* < 0.001). The turnover rate for the first year in the experimental group was significantly lower than the control group. The other two years were not significantly different.	Unknown	Unknown
Zhong, X. (2021) [84]	Nurses in the experimental group had significantly higher scores of professional identity and problem-solving ability (*p* < 0.001) than those in the control group.	The turnover intention of the nurses in the intervention group was significantly lower than the control group (*p* < 0.001). The scores of waiting to see a doctor, education of health knowledge, quality of nursing and the ward environment were significantly better in the intervention group (*p* < 0.001).	Unknown	Unknown

## Data Availability

The authors confirm that the data supporting the findings of this study are available within the article and/or its Supplementary Materials.

## Supplementary Materials

The following supporting information can be downloaded at: https://www.mdpi.com/article/10.3390/healthcare11131887/s1, S1: PRISMA Checklist and SWiM items; S2: Literature search; S3: Quality assessment MMAT.

## Abbreviations

ANA	American Nurses Association
AMSN	Academy of Medical-Surgical Nurses
APU	the Academic Partnership Program
ARCC	Advancing Research and Clinical practice through close Collaboration
BF	Bayes factor
BSN	Bachelor of Science in Nursing
CACP	the Corporate-Academic Cooperation Program
CDM	Care Delivery Model
CI	Confidence Interval
CPIRD	the Collaborative Project to Increase Production of Rural Doctor
CSE	Change-related Self-Efficacy
EBP	Evidence Based Practice
EERSO	Effort Ensuring Smooth Operation
EFQM	European Foundation for Quality Management
ENP	Emergency Nurse Physician
EP	Externship Program
FCP	Final Clinical Practicum
HDTS	Humanoid Diagram Teaching Strategy
HSP	High Preceptor Support
LSP	Low Preceptor Support
MEPRA	Mindful Ethical Practice and Resilience Academy
METEOR	MEnTal hEalth: fOcus on Retention of healthcare workers
MMAT	Mixed Methods Appraisal Tool
NA	Not Applicable
ODOD	One District One Doctor
OR	Odds Ratio
Prisma	Preferred Reporting Items for Systematic Review and Meta-Analysis
Prospero	International prospective register of systematic review
UNMCON	University of New Mexico College of Nursing
UNMH	University of New Mexico Hospital
SBAR	Situation, Background, Assessment, and Recommendation
SD	Standard Deviation
SITP	Situational Initiation Training Program
SWiM	Synthesis without meta-analysis
TeamSTEPPS	Team Strategies and Tools to Enhance Performance and Patient Safety
TSPP	Transition to Specialty Practice Program
TTP	Transition-To-Practice
