# Supplementary material for: Retaining Healthcare Workers: A Systematic Review of Strategies for Sustaining Power in the Workplace
Source: Healthcare (Basel). 2023 Jun 29;11(13):1887. doi: 10.3390/healthcare11131887 (PMC10341299; doi:10.3390/healthcare11131887)
Supplement: Supplementary file 1 [file healthcare-11-01887-s001.zip › healthcare-2454640-supplementary.pdf]

S1. PRISMA Checklist and SWiM items

Section/topic	#	Checklist item	Reported on page #
**TITLE**			
Title	1	Identify the report as a systematic review, meta-analysis, or both.	1
**ABSTRACT**			
Structured summary	2	Provide a structured summary including, as applicable: background; objectives; data sources; study eligibility criteria, participants, and interventions; study appraisal and synthesis methods; results; limitations; conclusions and implications of key findings; systematic review registration number.	2
**INTRODUCTION**			
Rationale	3	Describe the rationale for the review in the context of what is already known.	5
Objectives	4	Provide an explicit statement of questions being addressed with reference to participants, interventions, comparisons, outcomes, and study design (PICOS).	5
**METHODS**			
Protocol and registration	5	Indicate if a review protocol exists, if and where it can be accessed (e.g., Web address), and, if available, provide registration information including registration number.	6
Eligibility criteria	6	Specify study characteristics (e.g., PICOS, length of follow-up) and report characteristics (e.g., years considered, language, publication status) used as criteria for eligibility, giving rationale.	6
Information sources	7	Describe all information sources (e.g., databases with dates of coverage, contact with study authors to identify additional studies) in the search and date last searched.	6
Search	8	Present full electronic search strategy for at least one database, including any limits used, such that it could be repeated.	70 (supplemental 2)
Study selection	9	State the process for selecting studies (i.e., screening, eligibility, included in systematic review, and, if applicable, included in the meta-analysis).	7
Data collection process	10	Describe method of data extraction from reports (e.g., piloted forms, independently, in duplicate) and any processes for obtaining and confirming data from investigators.	7
Data items	11	List and define all variables for which data were sought (e.g., PICOS, funding sources) and any assumptions and simplifications made.	6

Risk of bias in individual studies	12	Describe methods used for assessing risk of bias of individual studies (including specification of whether this was done at the study or outcome level), and how this information is to be used in any data synthesis.	7
Summary measures	13	State the principal summary measures (e.g., risk ratio, difference in means).	N.A.
Synthesis of results	14	Describe the methods of handling data and combining results of studies, if done, including measures of consistency (e.g., I^2) for each meta-analysis.	N.A.

Section/topic	#	Checklist item	Reported on page #
Risk of bias across studies	15	Specify any assessment of risk of bias that may affect the cumulative evidence (e.g., publication bias, selective reporting within studies).	7
Additional analyses	16	Describe methods of additional analyses (e.g., sensitivity or subgroup analyses, meta-regression), if done, indicating which were pre-specified.	N.A.
**RESULTS**			
Study selection	17	Give numbers of studies screened, assessed for eligibility, and included in the review, with reasons for exclusions at each stage, ideally with a flow diagram.	8, 37 (fig 1)
Study characteristics	18	For each study, present characteristics for which data were extracted (e.g., study size, PICOS, follow-up period) and provide the citations.	38-45 (table 1), 46-65 (table 2)
Risk of bias within studies	19	Present data on risk of bias of each study and, if available, any outcome level assessment (see item 12).	N.A.
Results of individual studies	20	For all outcomes considered (benefits or harms), present, for each study: (a) simple summary data for each intervention group (b) effect estimates and confidence intervals, ideally with a forest plot.	N.A.
Synthesis of results	21	Present results of each meta-analysis done, including confidence intervals and measures of consistency.	N.A.
Risk of bias across studies	22	Present results of any assessment of risk of bias across studies (see Item 15).	N.A.
Additional analysis	23	Give results of additional analyses, if done (e.g., sensitivity or subgroup analyses, meta-regression [see Item 16]).	N.A.
**DISCUSSION**			
Summary of evidence	24	Summarize the main findings including the strength of evidence for each main outcome; consider their relevance to key groups (e.g., healthcare providers, users, and policy makers).	25

Limitations	25	Discuss limitations at study and outcome level (e.g., risk of bias), and at review-level (e.g., incomplete retrieval of identified research, reporting bias).	26,27
Conclusions	26	Provide a general interpretation of the results in the context of other evidence, and implications for future research.	27
**FUNDING**			
Funding	27	Describe sources of funding for the systematic review and other support (e.g., supply of data); role of funders for the systematic review.	28

From: Moher D, Liberati A, Tetzlaff J, Altman DG, The PRISMA Group (2009). Preferred Reporting Items for Systematic Reviews and Meta-Analyses: The PRISMA Statement. PLoS Med 6(6): e1000097. doi:10.1371/journal.pmed1000097

Section and Topic	Item #	Checklist item	Location where item is reported
**METHODS**			
Grouping studies for synthesis	1a	Provide a description of, and rationale for, the groups used in the synthesis (e.g., groupings of populations, interventions, outcomes, study design)	6
	1b	1b) Detail and provide rationale for any changes made subsequent to the protocol in the groups used in the synthesis	6 (prospero)
Describe the standardised metric and transformation methods used	2	Describe the standardised metric for each outcome. Explain why the metric(s) was chosen and describe any methods used to transform the intervention effects, as reported in the study, to the standardised metric, citing any methodological guidance consulted	N.A.
Describe the synthesis methods	3	Describe and justify the methods used to synthesise the effects for each outcome when it was not possible to undertake a meta-analysis of effect estimates	7
Criteria used to prioritise results for summary and synthesis	4	Where applicable, provide the criteria used, with supporting justification, to select the particular studies, or a particular study, for the main synthesis or to draw conclusions from the synthesis (e.g., based on study design, risk of bias assessments, directness in relation to the review question	6,7
Investigation of heterogeneity in reported effects	5	State the method(s) used to examine heterogeneity in reported effects when it was not possible to undertake a meta-analysis of effect estimates and its extensions to investigate heterogeneity	7,<br>28-45 (table 1),<br>46-65 (table 2)
Certainty of evidence	6	Describe the methods used to assess the certainty of the synthesis findings	7
Data presentation methods	7	Describe the graphical and tabular methods used to present the effects (e.g., tables, forest plots, harvest	7,

Section and Topic	Item #	Checklist item	Location where item is reported
		plots).<br>Specify key study characteristics (e.g., study design, risk of bias) used to order the studies, in the text and any tables or graphs, clearly referencing the studies included	28-45 (table 1), 46-65 (table 2)
**RESULTS**			
Reporting results	8	For each comparison and outcome, provide a description of the synthesised findings and the certainty of the findings. Describe the result in language that is consistent with the question the synthesis addresses, and indicate which studies contribute to the synthesis	9-24<br>38-45 (table 1), 46-65 (table 2)
**DISCUSSION**			
Limitations of the synthesis	9	Report the limitations of the synthesis methods used and/or the groupings used in the synthesis and how these affect the conclusions that can be drawn in relation to the original review question	26-27

From: Campbell M, McKenzie J E, Sowden A, Katikireddi S V, Brennan S E, Ellis S et al. Synthesis without meta-analysis (SWiM) in systematic reviews: reporting guideline BMJ 2020; 368 :l6890

S2. Literature search.

PubMed search

Domain	Determinants	Outcome
**Healthcare professionals**	**Interventions**	**Job retention/turnover**
"Health Personnel"[Mesh] OR "Health Personnel" OR "Healthcare workers" OR "healthcare providers" OR "healthcare professionals" OR "health workforce" OR "Health workers"	“Interventions”	"Personnel Turnover"[Mesh] OR "Personnel Turnover"
OR		OR
"Nurses"[Mesh] OR Nurse* OR "nursing personnel" OR "Physicians"[Mesh] OR “Physician” OR “doctor” OR "medical specialist"		"Retaining personnel" OR "job retention" OR "retention rates"
		OR

		"Turnover intention" OR "intention to leave" OR "intention to quit" OR "intention to stay"
		OR
		"Employee Turnover"

Filters: **from 2012 - 2022**

Embase Search

Domain	Determinants	Outcome
**Healthcare professionals**	**Interventions**	**Job retention**
exp health care personnel/	interventions	(Personnel-Turnover or retaining-personnel or job-retention or -retention-rates or turnover-intention or intention-to-leave or intention-to-quit or intention-to-stay or Employee-Turnover)[1]
OR		
exp nurse/		
OR		
exp physician/		
OR		

(Health-Personnel or Healthcare-workers or healthcare-providers or healthcare-professionals or health-workforce or Health-workers or Nurse* or nursing-personnel or Physician* or doctor* or medical-specialist)[1]		

[1] [mp=title, abstract, heading word, drug trade name, original title, device manufacturer, drug manufacturer, device trade name, keyword, floating subheading word, candidate term word]

CINAHL-search

Domain	Determinants	Outcome
**Healthcare professionals**	**Interventions**	**Job retention/turnover**
Health Personnel OR Healthcare workers OR healthcare providers OR healthcare professionals OR health workforce OR Health workers OR Nurses OR Nurse OR nursing personnel OR Physicians OR Physician OR Doctor OR medical specialist	interventions	Personnel Turnover OR retaining personnel OR job retention OR retention rates OR turnover intention OR intention to leave OR intention to quit OR intention to stay OR Employee Turnover

S3. Quality assessment MMAT[a]

First author	Year	S1	S2	1.1	1.2	1.3	1.4	1.5	2.1	2.2	2.3	2.4	2.5	3.1	3.2	3.3	3.4	3.5
**Adams, A.**	2019	Yes	Yes											Yes	Yes	Yes	No	Cannot tell
**Al Sabei, SD.**	2022	Yes	Yes															
**Alvaro, C.**	2016	Yes	Yes															
**Arora, R.**	2017	Yes	Yes											Cannot tell	Yes	Yes	No	Yes
**Aull, M.**	2022	Yes	Yes															
**Baik, D.**	2019	Yes	Yes											Yes	Yes	Yes	No	Yes
**Baillie, L.**	2019	Yes	Yes	Yes	No	Yes	Yes											
**Blegen, MA.**	2015	Yes	Yes											Yes	Yes	Yes	No	Cannot tell
**Brabson, LA.**	2019	Yes	Yes											Yes	Yes	Yes	Yes	Cannot tell
**Brewer, CS.**	2012	Yes	Yes											Yes	Yes	Cannot tell	No	Cannot tell
**Camveren, H.**	2022	Yes	Yes											Yes	Yes	Yes	No	Yes
**Chang, HY.**	2021	Yes	Yes											Yes	Yes	Yes	Yes	Yes
**Chen, S.**	2021	Yes	Yes															
**Chu, X.**	2022	Yes	Yes															
**Concilio, L.**	2021	Yes	Yes						Can not tell	Can not tell	No	Yes	Yes					
**Daniels, F.**	2012	Yes	Yes											Yes	Yes	Yes	No	Cannot tell
**Dawood, M.**	2019	Yes	Yes	Yes	Yes	Can not tell	Yes	Yes										

**Dawson, AJ.**	2014	Yes	Yes	Yes	Yes	Yes	No	No										
**Deng, J.**	2019	Yes	Yes															
**Duffield, C.**	2018	Yes	Yes											Yes	Yes	Yes	Yes	Yes
**Duru, DC.**	2022	Yes	Yes	No	No	Can not tell	No	No										
**El Khamali, R.**	2018	Yes	Yes						Yes	Yes	Yes	Yes	Yes					
**Fleig-Palmer, M.**	2015	Yes	Yes											Yes	No	Yes	Yes	Cannot tell
**Fleming, P.**	2012	Yes	Yes											Yes	Yes	Yes	No	No
**Forde-Johnston, C.**	2022	Yes	Yes															
**Gilroy, H.**	2020	Yes	Yes															
**Guo, YF.**	2020	Yes	Yes						Yes	Yes	Yes	Can not tell	Can not tell					
**Harris, KK.**	2017	Yes	Yes															
**Hernandez, SHA.**	2020	Yes	Yes											Yes	Yes	Yes	No	Yes
**Hines, M.**	2019																	
**Huang, TL.**	2022	Yes	Yes											Yes	Yes	Yes	Yes	Yes
**Im, SB.**	2016	Yes	Yes						Can not tell	Yes	Yes	Yes	Yes					
**Jensen, CL.**	2021	Yes	Yes						Yes	Yes	Yes	Yes	Yes					
**Joswiak, M.**	2018	No	Can not tell	Can not tell	Can nott tell	Can not tell	Can not tell	Can not tell										
**Kaihlanen, AM.**	2020	Yes	Yes											Yes	Yes	Can not tell	Yes	Yes
**Kang, CM.**	2016	Yes	Yes															

**Kang, J.**	2017	Yes	Yes															
**Kang, J.**	2019	Yes	Yes											Yes	Yes	Yes	Yes	Cannot tell
**Kester, KM.**	2020	Yes	Yes															
**Koneri, L.**	2021	Yes	Yes											Yes	Yes	Yes	Yes	No
**Kullberg, A.**	2016	Yes	Yes											Yes	Yes	Yes	No	Yes
**Melnyk, BM.**	2021	Yes	Yes											Yes	Yes	Yes	Yes	Yes
**Mohamadzadeh Nojehdehi, M.**	2015	Yes	Yes															
**Morphet, J.**	2015	Yes	Yes															
**Moss, M.**	2022	Yes	Yes											No	Yes	Yes	Yes	Yes
**Rudin, NMN.**	2018	Yes	Yes															
**Rushton, CH**	2021	Yes	Yes											Yes	Yes	Yes	Yes	Cannot tell
**Schroyer, CC.**	2020	Yes	Yes											Yes	Yes	Yes	Yes	Yes
**Tang, Y.**	2022	Yes	Yes											Yes	Yes	Yes	Yes	Yes
**Tseng, C. N.**	2013	Yes	Yes											Yes	Yes	Yes	No	Yes
**Vardaman, JM.**	2020	Yes	Yes											Can not tell	Yes	Yes	Cannot tell	Yes
**Walker-Czyz, A.**	2016	Yes	Yes											Can not tell	Yes	Can not tell	Yes	Cannot tell
**Williams, FS.**	2018	Yes	Yes											Yes	Yes	Yes	Yes	Cannot tell
**Winslow, S.**	2019	Yes	Yes											Can not tell	Yes	Can not tell	No	Cannot tell
**Wright, C.**	2017	Yes	Yes											Cannot tell	Yes	Can not tell	No	Yes
**Zhang, Y.**	2019	Yes	Yes											Yes	Yes	Yes	Yes	Yes
**Zhong, X.**	2021	Yes	Yes											Yes	Yes	Yes	No	Yes

First author	Year	4.1	4.2	4.3	4.4	4.5	5.1	5.2	5.3	5.4	5.5	Comments
**Adams, A.**	2019											
**Al Sabei**	2022	Yes	Yes	Yes	Yes	Yes						
**Alvaro, C.**	2016						Yes	Yes	Yes	Yes	Yes	
**Arora, R.**	2017											
**Aull, M.**	2022	Cannot tell	Cannot tell	Cannot tell	Cannot tell	Yes						
**Baik, D.**	2019											
**Blegen, MA.**	2015											
**Brabson, LA.**	2019											
**Brewer, CS.**	2012											
**Camveren, H.**	2022											
**Chang, HY.**	2021											
**Chen, S.**	2021	Yes	Yes	Yes	Cannot tell	Yes						
**Chu, X.**	2022	Yes	Yes	Yes	No	Yes						
**Concilio, L.**	2021											
**Daniels, F.**	2012											
**Dawood, M.**	2019											
**Dawson, AJ.**	2014											
**Deng, J.**	2019						Yes	Yes	Yes	Yes	Yes	
**Duffield, C.**	2018											
**Duru, DC.**	2022											
**El Khamali, R.**	2018											
**Fleig-Palmer, M.**	2015											
**Fleming, P.**	2012											
**Forde-Johnston, C.**	2022						No	Yes	Yes	N.A.	Yes	

**Gilroy, H.**	2020	No	Cannot tell	Yes	Yes	No						
**Guo, YF.**	2020											
**Harris, KK.**	2017						No	Cannot tell	Cannot tell	Cannot tell	Yes	
**Hernandez, SHA.**	2020											
**Hines, M.**	2019											
**Huang, TL.**	2022											
**Im, SB.**	2016											
**Jensen, CL.**	2021											
**Joswiak, M.**	2018											
**Kaihlanen, AM.**	2020											
**Kang, CM.**	2016						Yes	Yes	Yes	Yes	Yes	
**Kang, J.**	2017						Yes	Yes	Yes	Yes	Yes	
**Kang, J.**	2019											
**Kester, KM.**	2020	Yes	Yes	Yes	Cannot tell	Yes						
**Koneri, L.**	2021											
**Kullberg, A.**	2016											
**Melnyk, BM.**	2021											
**Mohamadzadeh Nojehdehi, M.**	2015	No	Yes	Yes	No	Yes						
**Morphet, J.**	2015						Yes	Yes	Yes	Yes	Yes	
**Moss, M.**	2022											
**Rudin, NMN.**	2018	Cannot tell	Yes	Yes	Cannot tell	Yes						
**Rushton, CH**	2021											
**Schroyer, CC.**	2020											
**Tang, Y.**	2022											
**Tseng, C. N.**	2013											

**Vardaman, JM.**	2020										
**Walker-Czyz, A.**	2016										
**Williams, FS.**	2018										
**Winslow, S.**	2019										
**Wright, C.**	2017										
**Zhang, Y.**	2019										
**Zhong, X.**	2021										

a. The complete questionnaire of the MMAT tool is available at http://mixedmethodsappraisaltoolpublic.pbworks.com/w/file/fetch/127916259/MMAT_2018_criteria-manual_2018-08-01_ENG.pdf
